# Letter to the Editor – Life cycle sustainability assessment without a life cycle?

**DOI:** 10.1007/s10661-023-11878-3

**Published:** 2023-09-20

**Authors:** Reinout Heijungs

**Affiliations:** 1 Department of Operations Analytics, Vrije Universiteit Amsterdam, De Boelelaan 1105, 1081 HV Amsterdam, The Netherlands; 2 Institute of Environmental Sciences, Leiden University, PO Box 9518, 2300 RA Leiden, The Netherlands

**Keywords:** Life cycle sustainability assessment (LCSA), Multi-criteria decision-making (MCDM), Life cycle, Landfill

A recent paper in this journal by Mozaffari et al. (2023) reported an analysis of the optimal location of a site for landfill operations in the city of Bardaskan, Iran. In their analysis, the authors claim to integrate life cycle sustainability assessment (LCSA) and GIS-based multi-criteria decision-making (MCDM). While we appreciate the authors’ appeal to GIS and MCDM, their idea of LCSA seems to be incorrect.

The authors correctly argue that LCSA is an integrated approach to cover the three aspects of sustainability: environmental, economic and social (Klöpffer, 2003, 2008). However, an equally indispensable characteristic of LCSA is its life cycle perspective. Mozaffari et al. (2023) cite Ren and Toniolo (2018) to prove that “traditional approaches of sustainability assessment have only concentrated upon one-step of the life cycle of the processes”. But this is not what Ren and Toniolo (2018) have in mind. In fact, those authors follow Klöpffer’s argument that a sustainability assessment should include more than an environmental analysis, covering the three pillars of sustainability. The life cycle of a product or system includes many steps, such as acquisition of raw materials, manufacture, transportation, use, maintenance and disposal. This is so, regardless the scope being environmental, economic, social, or a combination of the three.

If we have a closer look at the study by Mozaffari et al. (2023), we find none of the typical hallmarks of a life cycle perspective. To mention just a few of such characteristics:

- almost all life cycle studies rely on and cite the ISO 14040/14044 standards;
- a life cycle study is based on a functional unit;
- they are typically reported in terms of “goal and scope definition”, “inventory”, “characterisation”, “impact assessment”, “allocation”, “system boundary”, “unit process”, and similar terms.

All of this is missing in the study by Mozaffari et al. (2023). As such, we dare to doubt if the study is in any sense based on the idea of a life cycle.

Their study combines an analysis of the environmental, economic and social aspects of an activity (landfilling) at a location (Bardaskan). That is valuable. But an activity is not a life cycle (Heijungs & Cucurachi, 2021). The study by Mozaffari et al. (2023) could be extended to cover the life cycle, for instance following the framework of Hu et al. (2013). But right now, there is nothing life cycle-ish about it, hence any claim regarding LCSA is inappropriate.

## Data Availability

Not applicable.
